# Chronic pain and subjective memory problems are dynamic risk factors for functional decline: An ACTIVE longitudinal study^☆^

**DOI:** 10.1016/j.exger.2025.112891

**Published:** 2025-09-10

**Authors:** Tyler R. Bell, Cynthia Felix, Nathalie Gider, George W. Rebok, Michael Crowe, Karlene Ball, Gail Wallace, Sheila Black, Kelsey R. Thomas, Katie M. Wheeler, Felix J. Kollasserry, Jeanine M. Parisi, Caitlin A. Northcutt

**Affiliations:** a Department of Psychiatry, University of California San Diego, La Jolla, CA, USA; b Department of Psychiatry, University of Pittsburgh, PA, USA; c Department of Cognitive Science, University of California San Diego, La Jolla, CA, USA; d Department of Mental Health, Johns Hopkins Bloomberg School of Public Health, MD, USA; e Department of Psychology, University of Alabama at Birmingham, Birmingham, AL, USA; f Department of Psychology, University of Alabama, Tuscaloosa, AL, USA; g VA San Diego Healthcare System, San Diego, CA, USA; h School of Computing and Information, University of Pittsburgh, PA, USA; i Department of Epidemiology & Environmental Health, Kentucky Injury Prevention and Research Center, University of Kentucky, Lexington, KY, USA

**Keywords:** Chronic pain, Subjective memory, Cognitive function, Physical function, Social function

## Abstract

Chronic pain is highly prevalent in older adults and is associated with elevated risk for disability and dementia. Subjective memory problems (SMPs) - self-rated memory difficulties - are also common and may serve as early indicators of functional decline. This study examined associations between SMPs and cognitive, physical, and psychosocial functioning among older adults with and without chronic pain. Data were drawn from 2365 participants in the Advanced Cognitive Training for Independent and Vital Elderly (ACTIVE) study. Multilevel models assessed the impact of baseline and time-varying SMPs on cognitive performance (memory, reasoning, processing speed), physical function (mobility, grip strength, self-rated physical health), and psychosocial outcomes (depressive symptoms, social functioning). Chronic pain, defined as moderate-to-severe pain using the SF-36 Bodily Pain Scale, was tested as a moderator. At baseline, greater SMPs were associated with poorer cognitive performance (βs: −0.52 to −0.23). SMPs and chronic pain were independently linked to worse physical function, lower social functioning, and more depressive symptoms (βs: −0.94 to −0.34; 0.51 to 1.01). Chronic pain uniquely predicted worse mobility and reduced grip strength. Prospectively, SMPs interacted with chronic pain to predict steeper declines in reasoning and physical function. Concurrent increases in SMPs paralleled further declines in most cognitive, physical, and psychosocial domains. Findings suggest that SMPs are stronger predictors of certain outcomes among older adults with chronic pain. These results highlight the importance of jointly considering subjective memory problems and chronic pain to better identify individuals at heightened risk for accelerated functional decline in later life.

## 1. Introduction

Chronic pain is highly prevalent among older adults with 38.7 % reporting persistent pain and 27.8 % experiencing intermittent pain (Patel et al., 2013; Ritchie et al., 2023). There is considerable evidence that chronic pain is a major contributor to functional disability in later life as well as cognitive disorders such as dementia (Khalid et al., 2020; Makino et al., 2019; Tzeng et al., 2018). One possible early indicator of function decline, which is common among older individuals with chronic pain, is subjective memory problems (SMPs) - self-rated difficulties in recalling or learning new information (Jessen et al., 2020). These difficulties are often the early symptoms of dementia and are more frequently reported by people living with chronic pain (Mazza et al., 2018). However, the clinical significance of these SMPs in chronic pain populations remain unclear. What specific functional impairments do they reflect, and what future functional declines might they predict? Addressing these questions could shed light on the timing and development of functional decline among individuals with chronic pain.

It is clear that people living with chronic pain consistently report higher levels of SMPs, as early as midlife. This phenomenon is observed across various types of chronic pain, including chronic back pain, idiopathic neck pain, and fibromyalgia (Castel et al., 2021; Lenoir et al., 2018). These reports span both prospective and retrospective memory difficulties (Munoz and Esteve, 2005). In older adults from community samples, two studies have directly linked pain to SMPs. Montejo and colleagues found that current pain explained twice as much variance in SMPs (~12 %) compared to other health issues and sensory deficits (~6 %) (Montejo et al., 2020). Similarly, Westoby and colleagues reported that individuals with recent pain had a 2.49-fold increased likelihood of reporting cognitive complaints (Westoby et al., 2009). Our study seeks to expand on these findings by focusing specifically on the relationship between chronic pain and subjective memory difficulties as well as how they may interact to predict clinical outcomes in older adults.

Evaluating the clinical significance of SMPs in chronic pain populations requires examining multiple functional domains. Most studies to date have focused on cognitive performance outcomes, showing moderate associations between SMPs and measures of executive function and episodic memory (Baker et al., 2018; Castel et al., 2021; Lenoir et al., 2018). More notable are the associations between SMPs and depressive symptoms, with depressive symptoms contributing as much as 29 % of the variance in SMPs (Castel et al., 2021; McCracken and Iverson, 2001). Despite these insights, the cross-sectional nature of many studies limits our clinical understanding of how SMPs predict future functional decline. Moreover, the scope of these studies is often narrow, excluding other important domains of functioning. For example, social and physical functioning, both of which are heavily impacted by chronic pain and predictive of dementia, are also major correlates of SMPs and under investigated (Cosentino et al., 2018; Kuiper et al., 2017).

The aim of this study is to investigate baseline, prospective, and longitudinal concurrent associations between SMPs and cognitive, social, and physical functioning in older adults with and without chronic pain, using data from the Advanced Cognitive Training for Independent and Vital Elderly (ACTIVE) study. ACTIVE is a multisite, randomized clinical trial involving adults aged 65 years and older who underwent extensive cognitive testing over a 12-year period. Although there is prior research from the ACTIVE study focusing on chronic pain and cognitive decline (Bell et al., 2022a, 2022b), our study will incorporate new data on SMPs alongside measures of physical, social, and emotional health, providing a more comprehensive understanding of how chronic pain may influence functional trajectories in older adulthood.

Regarding hypotheses, a prevailing theoretical framework posits that SMPs may contribute to functional decline through behavioral and psychosocial pathways. Specifically, individuals who perceive memory difficulties may curtail participation in cognitively, socially, and physically engaging activities, thereby accelerating deterioration in these domains over time (Lee and Foster, 2023). Alternatively, other functional challenges - such as emotional distress or physical limitations - may lead individuals to overgeneralize their health concerns, interpreting them as indicative of broader cognitive decline and reporting greater SMPs.

Building on this framework, our specific hypothesis is that chronic pain exacerbates SMPs-associated functional decline, particularly in reasoning and self-rated physical function. Chronic pain may heighten the impact of SMPs through multiple, interacting mechanisms. First, persistent pain places continuous demands on attentional resources, diverting cognitive capacity away from executive control processes such as reasoning (Moore et al., 2012). Second, pain-related fear and avoidance behaviors can reduce engagement in cognitively and physically stimulating activities, accelerating deconditioning and functional limitations (Martinez-Calderon et al., 2019; Zhaoyang et al., 2020). Over time, these processes may create a self-reinforcing cycle in which pain, SMPs and reduced activity interact to hasten declines in both cognitive and physical domains.

## 2. Method

### 2.1. Design and participants

This study utilized data from the Advanced Cognitive Training for Independent and Vital Elderly (ACTIVE, ClinicalTrials.gov Identifier NCT00298558) randomized controlled trial. Conducted at six sites across the United States, the ACTIVE trial investigated the effectiveness of three cognitive training programs (compared to a no-contact control) on cognition and daily functioning in older adults. Participants were recruited from March 1998 to October 1999 and included individuals aged 65 and older who did not meet the following exclusion criteria: (1) potential cognitive impairment (MMSE <23 or reported dementia diagnosis), (2) functional impairment (requiring extensive assistance with activities of daily living), (3) self-reported stroke in the last 12 months, (4) certain cancer diagnoses or current chemo- or radiation therapy, (5) vision, hearing, or communication problems hindering participation, (6) prior cognitive training, or (7) inability to meet study time requirements. Additional details about the ACTIVE trial can be found elsewhere (Jobe et al., 2001).

The ACTIVE study had a total sample size of 2802, which was designed to achieve 95 % power to detect a Cohen’s d effect size of 0.20 for training transfer effects (Ball et al., 2002). Among these participants, 2365 had data on bodily pain at the first and second assessment, forming our main analytical sample. Data availability for study outcomes is listed in Table 1 by chronic pain status. The analytic sample had a mean baseline age of 73.20 years (SD = 5.66, range: 65, 94), was predominantly female (73 %), White (75 %), and widowed or single (60 %). On average, participants reported 13.70 years of education (SD = 2.68, range: 6 to 20 years). Additional sample characteristics are presented in Table 1.

### 2.2. Procedure

All study procedures were approved by the Institutional Review Boards at participating sites. After an initial telephone screening, eligible participants provided written informed consent in person and completed a baseline assessment including surveys and evaluations of daily habits, psychosocial functioning, health status, and physical and cognitive performance. Participants were then randomized to one of three cognitive training groups or a no-contact control group. The ACTIVE intervention targeted memory, reasoning, and speed of processing - domains known to show early age-related decline and to be closely tied to daily functioning. Training was delivered in small groups over ten 60–75-min sessions across 5–6 weeks. Memory training emphasized verbal episodic memory through instruction and practice in mnemonic strategies; reasoning training focused on solving problems with serial patterns; and speed-of-processing training enhanced visual search and the capacity to manage increasingly complex information presented in progressively shorter time intervals. Booster training, consisting of four 75-min sessions at 11- and 35-months post-intervention, was offered to a random subset (39 %) of participants in each training group who had completed at least eight of the ten initial sessions. Follow-up assessments occurred immediately post-intervention and at 1, 2, 3, 5, and 10 years. The present study included participants from all intervention conditions, adjusting for intervention effects in analyses to maximize sample size. All data relevant to this publication are publicly available (https://www.icpsr.umich.edu/web/NACDA/studies/36036) as is the Syntax for data preparation and data analyses. (https://github.com/trbellucsd/ACTIVE_Pain_Subjective_Memory).

### 2.3. Measures

#### 2.3.1. Chronic pain

Pain was assessed using the SF-36 Quality of Life questionnaire version 1 (Ware Jr. and Sherbourne, 1992). Pain severity is explicitly asked as “How much pain severity have you had during the past 4 weeks?” Participants answer on a 6-point Likert-type scale selecting “None” (1), “Very Mild” (2), “Mild” (3), “Moderate” (4), “Severe” (5), “Very severe” (6). Research has shown that this SF-36 pain item correlates well with comprehensive pain scales, such as the Brief Pain Inventory (BPI) and the McGill Pain Questionnaire, supporting construct validity (rs ranges from 0.76 to 0.79) (Gandek et al., 2004; Jensen et al., 1999; Ware Jr. and Sherbourne, 1992). We specifically defined chronic pain based on moderate-to-severe levels reported at both baseline and the first follow-up on this item (one year apart). This definition is consistent with the definition of chronic pain from the International Association for the Study of Pain, which defines chronic pain as pain persisting or reoccurring for over 3 or more months (Treede et al., 2019). The designation of chronic pain based on moderate-to-severe pain severity aligns with studies considering these levels to be clinically significant (Chow et al., 2016; Von Korff et al., 2020). While not based on formal clinical diagnosis, this measure of chronic pain has shown comparable findings to clinical diagnosis when assessing cognitive decline and dementia risk (Bell et al., 2025; Bell et al., 2022a, 2022b; Khalid et al., 2020; Whitlock et al., 2017).

#### 2.3.2. Subjective memory problems

Subjective memory was assessed using the Memory Failures Questionnaire General Frequency of Forgetting subscale (MFQ-GFF) (Gilewski et al., 1990). The original MFQ-GFF subscale includes 14 items that represent memory problems. Examples include trouble remembering phone numbers, things people tell you, going to the store and forgetting what to buy, and trouble remembering what you have read. Participants were asked to rate how often these were a problem for them on a scale from 1 (always) to 7 (never), such that higher scores represent better subjective memory. The total scale had high reliability in the study (alpha = 0.92). The scale was reverse scored for the current study so that higher scores indicated higher SMPs.

For figure illustration, we used a median-split (median = 24.00) to designate high versus low SMP for easy visualization.

#### 2.3.3. Cognitive function

Cognitive performance was assessed across several domains, including speed of processing, memory, and reasoning. Speed of processing was assessed using the Useful Field of View (UFOV^®^) test (Ball and Owsley, 1993). This was calculated as a z-score composite UFOV^®^ subtest 2 (UFOV2), UFOV^®^ subtest 3 (UFOV3), and UFOV^®^ subtest 4 (UFOV4). UFOV2 required identifying a central object and locating a peripheral object. UFOV3 involved identifying the central object and locating the peripheral object with distractor symbols included, while UFOV4 included a more complex central identification task along with the peripheral localization task and distractors. Episodic memory was evaluated using accuracy recall scores on the Hopkins Verbal Learning Test (HVLT) (Brandt, 2001), Rey-Auditory Verbal Learning Test (AVLT) (Lezak, 2004), and the Paragraph Recall subtest (PRT) of the Rivermead Behavioral Memory Test (Wilson and Baddeley, 1985). These tests required participants to listen to a list of words or a short story and immediately recall the information, with scores reflecting the total number of correctly recalled words. Reasoning abilities were measured based on accuracy on tasks from the Adult Development and Enrichment Project, specifically the Letter Series, Letter Sets, and Word Series tasks (Blieszner et al., 1981). Both tasks involve executive function and problem-solving skills to identify patterns of letters or words. For composites, test performance was z-scored then used to create an average z-score for each cognitive domain. All tests and their resulting composites were z-scored in a direction such that more positive scores indicated better performance.

#### 2.3.4. Physical function

Self-rated physical function was measured using the SF-36 Questionnaire that was administered at all waves (McHorney et al., 1993). Participants were asked to rate various activities “1 - Yes, limited a lot,” “2, Yes, limited a little,” and “3, No, not limited at all.” Activities included (1) vigorous activities such as running, lifting heavy objects, and participating in strenuous sports, (2) moderate activities such as moving a table, pushing a vacuum, bowling, or playing golf, (3) ability to lift or carry groceries, (4) climb several flights of stairs, (5) climb one flight of stairs, (6) bending, kneeling, or stooping, (7) walking more than a mile, (8) walking several blocks, (9) walking one block, (10) bathing or dressing yourself. This measure showed high internal reliability and studies have shown strong associations with objective measures of physical function (McHorney et al., 1993; Ware & Sherbourne, 1992).

Objective physical function was assessed using the Turn360 test and grip strength. The Turn360 task examines balance and mobility by asking participants to make a full 360-degree turn in place, to either the right or the left. Total steps to complete the turn is scored, with more steps indicating worse physical performance. People who were not able to complete the Turn360 task due to physical inability were imputed with the highest number of steps shown by participants completing the task. Grip strength was measured using a handheld dynamometer that captures the maximum force (kg) exerted by the hand for a few seconds. Participants used both their dominant and non-dominant hands, holding the dynamometer with their elbow bent at a 90-degree angle. The maximum kg for the left and right hand were averaged together as the final grip strength measure. Participants unable to complete the task due to physical inability were imputed with the lowest grip strength shown by participants completing the task.

#### 2.3.5. Psychosocial function

Baseline depressive symptoms were assessed using the total score of the Center for Epidemiologic Studies Depression Scale-12 item (CESD) (Radloff, 1977). The CESD asks participants to indicate how often they experienced 12 depressive symptoms during the previous week, with response options ranging from none of the time (0) to most or all the time (3). Higher CESD scores indicate more severe depressive symptoms.

Social function refers to an individual’s ability to engage in, maintain, and navigate interpersonal relationships and social roles (World Health Organization, 2016). Social function was determined by one item from the SF-36 (McHorney et al., 1993). Participants were asked “During the past 4 weeks, to what extent has your physical health or emotional problems interfered with your normal social activities with family, friends, neighbors, or groups” rated on a five-point Likert-type scale from 1, Not at all to 5, Extremely. Their response was taken to determine level of social function.

#### 2.3.6. Covariates

We adjusted for sociodemographic factors commonly included in pain studies (Whitlock et al., 2017). These variables consisted of time-invariant factors such as baseline age (measured in years), race (coded as White 1; non-White 0), sex (coded as male 1; female 0), years of formal education (ranging from 0 for no education to 20 for doctoral-level or equivalent), and marital status (coded as married/partnered 1; separated/widowed 0).

### 2.4. Analysis plan

For descriptive analyses, we examined frequency and correlates of SMPs in people with and without chronic pain (see Table 1). For our main analyses, we constructed multilevel models to examine the association of baseline SMPs and time-varying SMPs (longitudinal changes in SMP over time) on functional outcomes including cognitive (speed, episodic memory, and reasoning) and non-cognitive outcomes (depressive symptoms, physical function, and social function). Our models additionally examine the moderating effect of chronic pain and controlled for sociodemographic covariates.

Data were analyzed in R version 4.2 using the “lmer” package. Analyses were conducted in R version 4.2 using the *lmer* package. Using package “simr” in R version 4.0 (Green & MacLeod, 2016), our sample had a 91 % power to detect even a small effect (*d* = 0.20) using mixed linear models. This high sensitivity increases our ability to detect even modest associations that may be clinically relevant. Missing data were handled using full information maximum likelihood (FIML), which uses all available observations for each participant under the assumption of missing at random (MAR). This approach retains participants with partial data, maximizing statistical power and reducing bias compared to listwise deletion (Baraldi and Enders, 2010). To ensure transparency, Table 1 reports the sample sizes available for each variable. The basic equation for the mixed model is as follows:

$$\text{Level 1}:{\text{Y}}_{\text{ij}}[\text{Functional Outcome}]=\text{γ}00+\text{β}1\left({\text{Time Since Baseline}}_{\text{ij}}\right)+\text{β}2\left(\text{Time}-\text{Varying SMPs}\right)+\text{β}3\left({\text{Baseline SMPs}}_{\text{i}}\right)\;\;+\text{β}4\left(\text{Time}-\text{Varying Depressive Symptoms}\right)+\text{β}5\left({{\text{Intervention Group}}_{\text{i}}}^{*}\text{Time Since Baseline}\right)\;\;+\text{β}6\left({{\text{Chronic Pain}}_{\text{i}}}^{*}{\text{Time Since Baseline}}_{\text{ij}}\right)+\text{β}7\left({{\text{Time}-\text{Varying SMPs}}_{\text{ij}}}^{*}{\text{Chronic Pain}}_{\text{i}}\right)\;\;+\text{β}8\left({{\text{Baseline SMPs}}_{\text{i}}}^{*}{\text{Time Since Baseline}}^{*}{\text{Chronic Pain}}_{\text{i}}\right)+{\text{e}}_{\text{ij}}$$

$$\text{Level 2}:{\text{β}0}_{\text{i}}[\text{Baseline Value of Functional Outcome}]=\text{γ}00+\text{γ}01\left({\text{Baseline Age}}_{\text{i}}\right)+\text{γ}02(\text{Chronic Pain})+\text{γ}03\left({\text{Baseline SMPs}}_{\text{i}}\right)\;\;+\text{γ}04\left({\text{Intervention Group}}_{\text{i}}\right)+\text{γ}05\left({\text{Race}}_{\text{i}}\right)+\text{γ}06\left({\text{sex}}_{\text{i}}\right)+\text{γ}07\left({\text{Education}}_{\text{i}}\right)\;\;+\text{γ}08\left({\text{Marital Status}}_{\text{i}}\right)+\text{γ}09\left({\text{*Baseline SMPs}}_{\text{i}}*{\text{Chronic Pain}}_{\text{i}}\right)+{\text{μ}}_{\text{i}}$$

where Y_i_j represents the functional outcome of interest for an individual (i) at each time point (j), β0_i_ is the baseline score of the functional outcome for each individual, γ00 is the sample mean of the functional outcome and β1 reflects the yearly rate of change in functional outcomes scores tested by our time variable of years after baseline (age at follow-up-age at baseline). Associations are reported in standardized betas (β), which are interpretable as effect sizes similar to Cohen’s *d* but specifically representing standard deviation differences in the outcome due to a level difference in a categorical variable (e.g., having chronic pain versus not) or a standard deviation unit increase in a continuous variable (MFQ-FF score ~ 14, or rating “always” on two additional memory problem items). Generally, for cognition and other health outcomes, values around 0.20 are considered small, meaning statistically meaningful but likely not involving observable differences/change in everyday life; while values around 0.30 to 0.50 are considered moderate and representing a minimally clinically important difference (Howard et al., 2011; Norman et al., 2003; Watt et al., 2021). To aid interpretation, we apply qualitative labels based on standardized coefficients: “slightly associated” for small effects (β ≈ 0.20), “moderately associated” for effects that are minimally clinically meaningful (β ≈ 0.40), and “substantially associated” for larger effects (β ≈ 0.80). As a note, 0.20 is common in cognitive interventions (Mewborn et al., 2017). Significance is determined using an alpha of 0.05.

## 3. Results

### 3.1. Sample descriptives

As shown in Table 1, approximately 16 % of our sample had chronic pain (*n* = 382). These individuals were more likely to be female sex, have fewer years of formal education, more depressive symptoms, and more SMPs compared to those without chronic pain. Regarding cognitive performance, people with chronic pain had lower social function and physical function on the SF-36 as well as worse performance on the Turn360 and grip strength tasks compared to those without chronic pain. Overall, higher SMPs in the sample were related to older age, White race, female sex, fewer years of formal education, more depressive symptoms, worse cognitive function (for all three composite scores), worse social function, and worse physical function.

### 3.2. Baseline associations

#### 3.2.1. Subjective memory problems

Baseline associations between SMPs and various functional outcomes are presented in Tables 2 to 4 (see Baseline Associations). Regarding cognitive outcomes, individuals who reported higher SMPs had a moderately lower memory performance (β = −0.47, 95 % CI: −0.64 to −0.40, *p* < .001), moderately reduced reasoning ability (β = −0.52, 95 % CI: −0.75 to −0.29, *p* < .001), and a slightly slower processing speed at baseline (β = −0.23, 95 % CI: −0.44 to −0.01, *p* = .037). These associations were similar across those with and without chronic pain (*p*s > 0.05).

In terms of physical function, baseline SMPs were moderated associated with moderately poorer self-rated physical function (β = −0.49, 95 % CI: −0.67 to −0.31, *p* < .001) and moderately reduced grip strength (β = −0.46, 95 % CI: −0.82 to −0.10, *p* = .012). However, no significant relationship was found between baseline SMPs and Turn360 task performance (*p*s > 0.05). These associations were similar across those with and without chronic pain (*p*s > 0.05). In practical terms,

For psychosocial outcomes, higher baseline SMPs was associated with moderately worse social function (β = −0.34, 95 % CI: −0.58 to −0.11, *p* = .004) and substantially higher depressive symptoms (β = 1.01, 95 % CI: 0.79 to 1.24, *p* < .001). These associations were consistent across individuals with and without chronic pain (*p*s > 0.05).

#### 3.2.2. Chronic pain

In these models, chronic pain was not related to differences in baseline cognitive function (*p*s > 0.05). However, chronic pain was also associated with substantially worse self-rated physical function (β = −0.94, 95 % CI: −1.03 to −0.86, *p* < .001), slightly greater steps needed to complete the Turn360 task (β =0.24, 95 % CI: 0.15 to 0.34, *p* < .001), and slightly worse grip strength at baseline (β = −0.15, 95 % CI: −0.30 to −0.01, *p* = .032). Chronic pain was independently associated with moderately worse social function (β = −0.55, 95 %CI: −0.62 to −0.48, *p* < .001) and moderately greater depressive symptoms at baseline (β = 0.51, 95 % CI: 0.43 to 0.59, *p* < .001).

### 3.3. Prospective associations

#### 3.3.1. Subjective memory problems

**Fig. 1.**
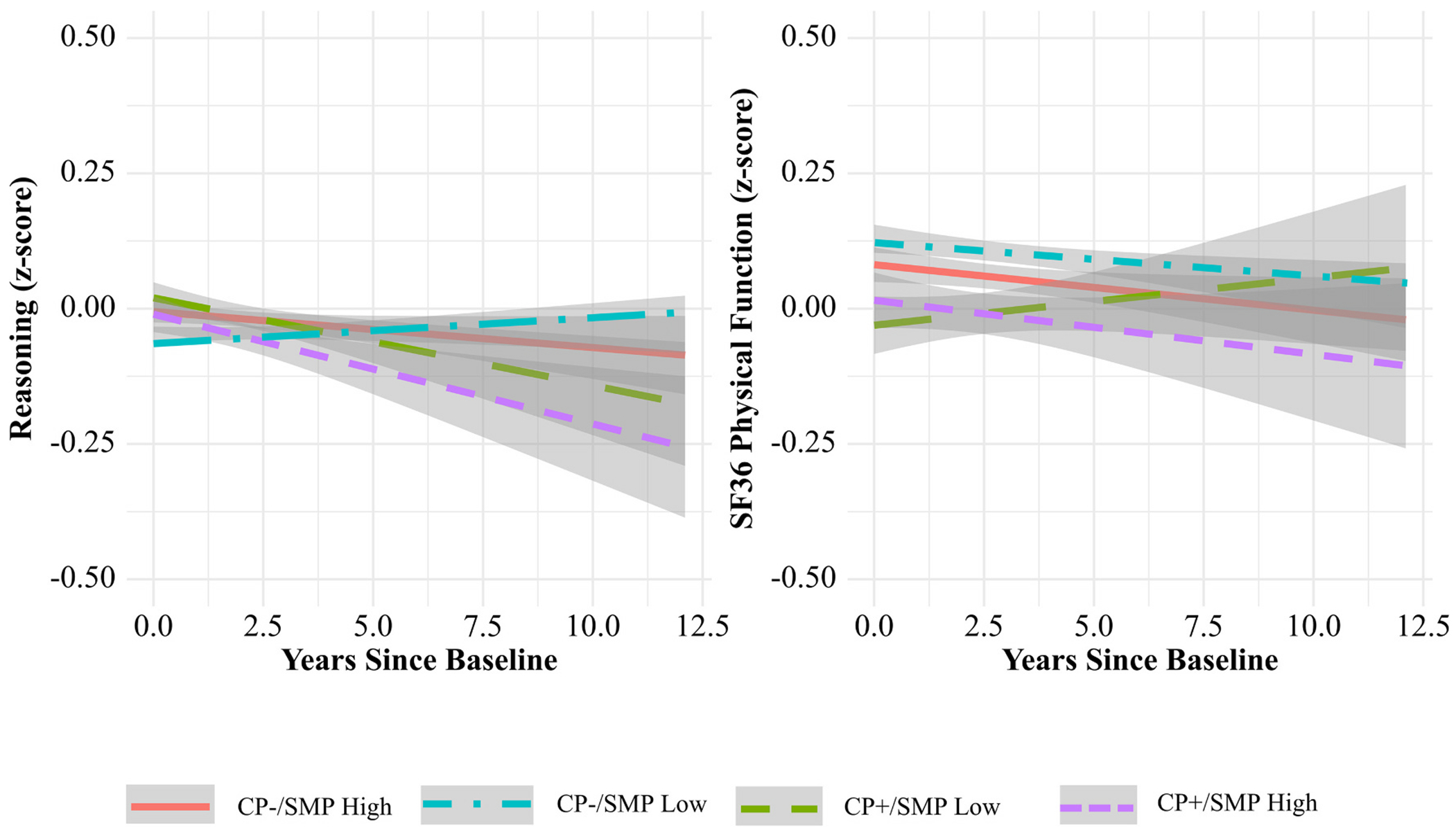
Interactive associations of subjective memory problems and chronic pain with reasoning and self-rated physical function. Note. CP+ = chronic pain present; CP− = no chronic pain; SMP Low = low level of subjective memory problems indicated as scoring below the median (median = 24.00); SMP High = high level of subjective memory problems indicated by scoring above the median (median = 24.00).

Prospective associations between baseline SMPs and functional outcomes are detailed in Tables 2 through 4 (see Prospective Associations). For cognitive changes, higher baseline SMPs was associated with a slightly greater decline in reasoning (β = −0.10, 95 % CI: −0.12 to −0.08, *p* < .001) but was unassociated with the rate of change in memory or processing speed (*p*s > 0.05). Furthermore, the association of higher SMPs with slightly steeper reasoning decline was accentuated among people with chronic pain (baseline SMPs*time*chronic pain: β = −0.05, 95 %CI: −0.09 to −0.01, *p* = .039). Fig. 1 shows this illustration while using a median-split (median = 24.00) to designate high versus low SMP for easy visualization.

**Fig. 2.**
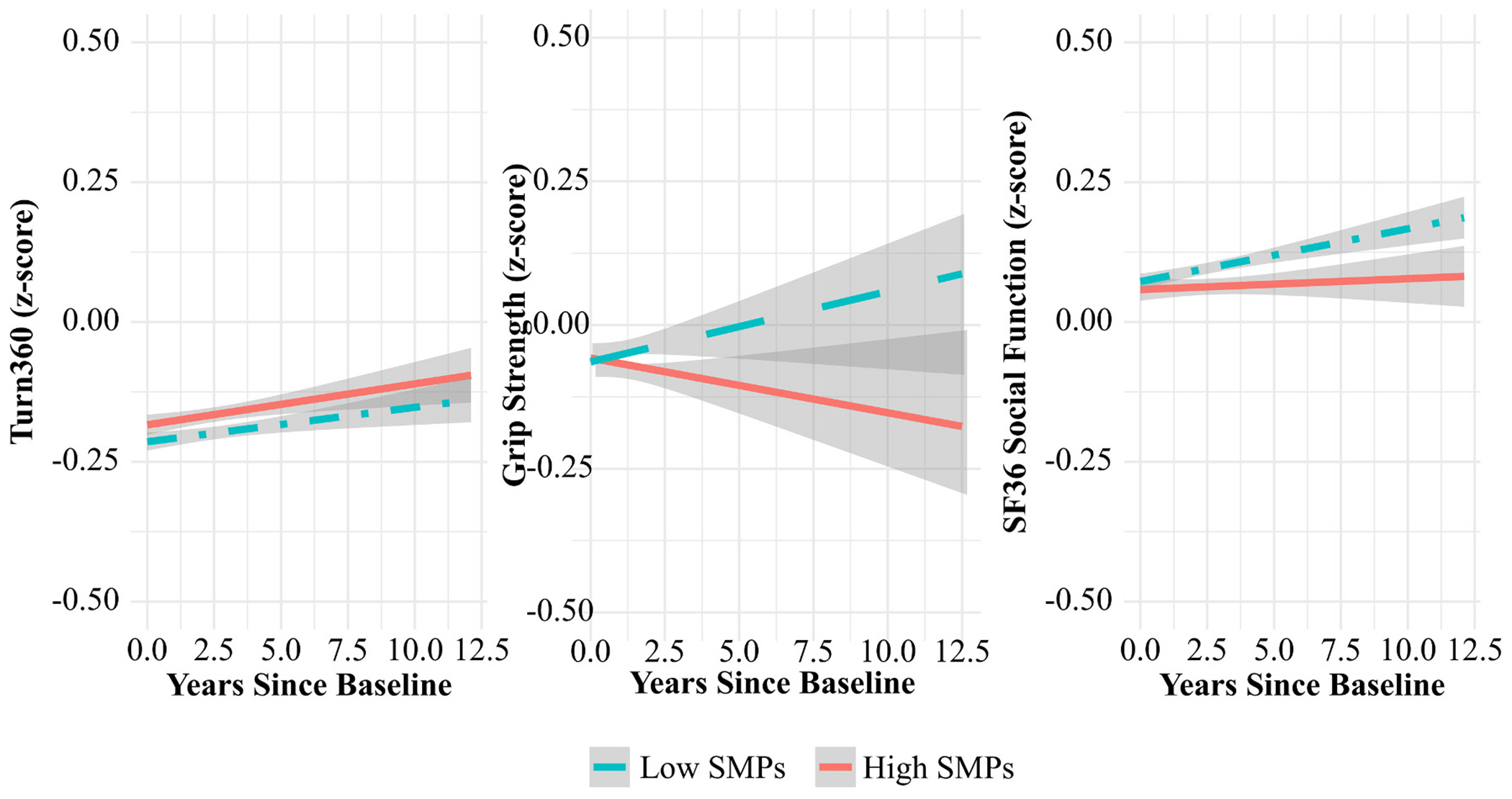
Independent associations of subjective memory problems with physical and social function. Grip strength = based on a dynamometer reading on the main hand used; SF-36 Social Function – subscale of the Short-Form 36 quality of life scale; SMP Low = low level of subjective memory problems indicated as scoring below the median (median = 24.00); SMP High = high level of subjective memory problems indicated by scoring above the median (median = 24.00); Turn360 = a mobility task where higher scores indicate more steps needed to turn around.

In terms of physical function, higher baseline SMPs was associated with a slightly steeper decline in self-rated physical function (β = −0.02, 95 % CI: −0.03 to −0.01, *p* = .008), slightly higher increase in the number of steps required to complete the Turn360 task (β = 0.03, 95 % CI: 0.01 to 0.05, *p* = .023), and a slightly steeper decline in grip strength (β = −0.10, 95 % CI: −0.15 to −0.04, *p* < .001). The association between higher baseline SMPs and slightly steeper decline in self-rated physical function was accentuated among people with chronic pain (β = −0.05, 95 %CI: −0.09 to −0.02, *p* = .001, see Fig. 1). Independent associations of baseline SMPs with rapid decline in Turn360 performance and grip strength are shown in Fig. 2. Fig. 2 shows this illustration while using a median-split (median = 24.00) to designate high versus low SMP for easy visualization.

Regarding psychosocial outcomes, greater baseline SMPs were associated with a slightly steeper decline in social function (β = −0.02, 95 % CI: −0.04 to −0.001, *p* = .027), which is illustrated in Fig. 2. Baseline SMPs were not related to changes in depressive symptoms over time (*p* = .693). These associations were similar for individuals with and without chronic pain (baseline SMPs*Time*Chronic Pain: *p*s > 0.05).

#### 3.3.2. Chronic pain

**Fig. 3.**
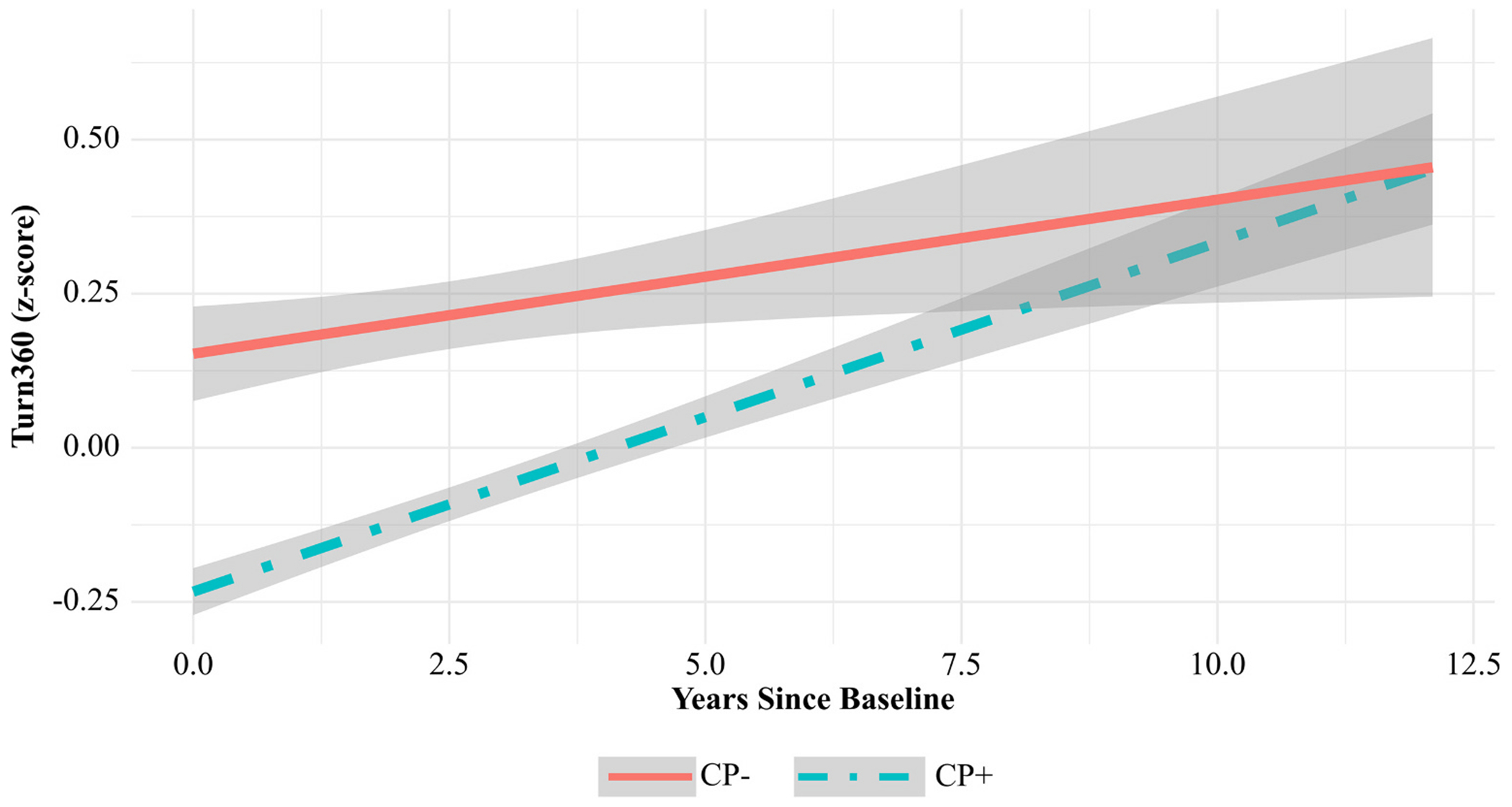
Association of chronic pain with change in Turn360 performance. Note. CP+ = chronic pain present; CP− = no chronic pain; Turn360 = a mobility task where higher scores indicate more steps needed to walk and turn around.

Chronic pain did not relate to the rate of change in memory or speed of processing (*p*s > 0.05). Regarding physical function, chronic pain was associated with a slightly less decline in steps needed to complete the Turn360 task (β =−0.06, 95 % CI: −0.11 to −0.02, *p* =.010), illustrated in Fig. 3. Chronic pain was unrelated to the rate of change grip strength (*p*s > 0.05). Regarding psychosocial outcomes, chronic pain was unrelated to changes in social function or depressive symptoms (*p*s > 0.05).

### 3.4. Concurrent longitudinal associations

Concurrent associations between changes in SMPs and changes in functional outcomes are presented in Tables 2 to 4 (see Concurrent Longitudinal Associations). Regarding cognitive function, increases in SMPs were moderately associated with declines in memory (β = −0.28, 95 % CI: −0.44 to −0.12, *p* = .001) and reasoning (β = −0.38, 95 % CI: −0.60 to −0.15, *p* = .001), but unrelated to changes in speed of processing (*p* = .208). The associations between time-varying SMPs and cognitive function were consistent across individuals with and without chronic pain (Time-Varying SMPs*chronic pain: *p*s > 0.05).

In terms of physical function, increases in SMPs were associated with moderate reductions in self-rated physical function (β = −0.35, 95 %CI: −0.52 to −0.18) and moderate reductions in grip strength (β = −0.37, 95 % CI: −0.72 to −0.03, *p* = .033). Change in SMPs were unrelated to Turn360 performance (*p* = .982). These associations were comparable between older adults with and without chronic pain (*p*s > 0.05).

Regarding psychosocial outcomes, increases in SMPs were associated with moderate reduction in social functioning (β = −0.26, 95 %CI: −0.49 to −0.03, *p* = .026) and substantially greater depressive symptoms (β = 0.80, 95 % CI: 0.58 to 1.02, *p* < .001). These associations were consistent across individuals with and without chronic pain (Time-Varying SMPs*Chronic Pain: *p*s > 0.05).

## 4. Discussion

The present study offers valuable insights into the relationship between SMPs and functional outcomes in older adults, with a particular focus on those experiencing chronic pain. Chronic pain is a widespread issue among older adults and has significant implications for both cognitive and physical health. Prior research has identified chronic pain as a significant risk factor for dementia, a condition marked by progressive cognitive and functional decline - including declines in physical and psychosocial function (Jutkowitz et al., 2017; Martyr et al., 2024). Moreover, SMPs have been suggested as early indicators of cognitive decline (Numbers et al., 2020), and may be useful to identify people with chronic pain who are at higher risk of negative health outcomes such as dementia (Westoby et al., 2009). Our study had notable findings, including the association between higher levels of SMPs and poorer cognitive performance, greater physical limitations, and reduced social functioning over time. Chronic pain served as both an additive risk factor and an exacerbating risk factor for functional decline when combined with SMPs. Findings underscore the complex interplay between chronic pain and subjective memory in shaping the trajectory of cognitive, physical, and psychosocial health in older adults.

Baseline analyses revealed that individuals with higher SMPs performed moderately worse in memory and reasoning as well as slightly slower on processing speed. Moreover, increases in SMPs were associated with moderate declines in memory and reasoning, a pattern that held true regardless of chronic pain status. However, prospective analyses uncovered group differences: higher baseline SMPs predicted slightly steeper declines in reasoning, particularly among individuals with chronic pain. This finding suggests that elevated SMPs, which may partly reflect elevated depression and anxiety (Castel et al., 2021; Jennifer et al., 2024), could also indicate the additional attentional demands imposed by chronic pain (Eccleston and Crombez, 1999; Moore et al., 2012). Since reasoning relies more heavily on attention than memory or processing speed, individuals with chronic pain and elevated SMPs may be less capable of compensating for attentional deficits, leading to accelerated reasoning decline. Previous research has shown that executive function tasks, like reasoning, are more sensitive to the attentional demands of chronic pain (Bell et al., 2022a, 2022b; Berryman et al., 2014; Moriarty et al., 2011; Whitlock et al., 2017). Future experimental work using objective markers of attention load may elucidate such a mechanism (e.g., pupillometry, EEG, fMRI). Regardless, results underscore that SMPs are more related to reasoning decline in people with chronic pain than those without chronic pain.

Regarding objective measures of physical function, our study found that SMPs were moderately associated with reduced mobility - as measured by the Turn360 task - and lower grip strength at baseline. Furthermore, increases in SMPs were associated with moderate reductions in grip strength. Prospectively, higher baseline SMPs predicted slightly worsening mobility over time based on Turn360 performance. Importantly, these associations held regardless of chronic pain status, though they appeared to add to the independent impact of chronic pain - consistent with prior research showing a direct association of chronic pain on these outcomes (Cruz-Almeida et al., 2017; Eggermont et al., 2014; Feldman and Nahin, 2022). Interestingly, chronic pain at baseline was also slightly linked to poorer grip strength and reduced Turn360 task performance. Notably, individuals with chronic pain exhibited a slower rate of mobility decline over time, likely because chronic pain had already significantly compromised their physical abilities, leaving less room for further decline therefrom (e.g., floor effect). Overall, these findings suggest that SMPs and chronic pain represent distinct yet interrelated challenges to physical function.

Results were different when looking at self-rated physical function. At baseline, SMPs and chronic pain independently related to worse self-rated physical function; and concurrent increases in SMPs related to decreases in self-rated physical function - comparable across people with and without chronic pain. Prospectively, SMPs and chronic pain interacted to predict changes in self-rated physical function. Higher baseline SMPs related to greater declines in self-rated physical function, and this association was magnified among people with chronic pain. These findings may occur because self-rated physical function captures not only objective abilities but also an individual’s perceptions, expectations, and emotional responses to their health. SMPs may reflect a broader sense of health decline undetected by objective physical performance, which influences how individuals perceive their overall function. When combined with chronic pain - which often leads to a persistent awareness of poorer health - the subjective experience of diminished function may be amplified. In essence, while chronic pain may predominantly impact objective measures like grip strength and mobility, SMPs might better capture declines in activities and daily functioning as perceived by the individual, particularly when chronic pain heightens these negative perceptions.

The psychosocial findings from our study add another layer of complexity to our understanding of SMPs, particularly in individuals with chronic pain. Consistent with prior research (Hill et al., 2020), higher levels of SMPs were associated with more depressive symptoms at baseline; and increases in SMPs were associated with increases in depressive symptoms over time. Baseline associations are consistent with prior research (Hill et al., 2020; Hill et al., 2016). These associations were comparable among older adults with and without chronic pain. This again suggests that SMPs serve as an independent risk factor for this outcome, although more likely to co-occur in people with chronic pain. Interestingly, despite these associations baseline SMPs did not predict increases in depressive symptoms over time. This unexpected finding may be explained by the fact that SMPs reported in younger ages are heavily influenced by a stable trait from early adulthood reflecting general negative affectivity (Bell et al., 2023; Hill et al., 2020). Meanwhile, due to new experiences in later life, changes in SMPs as one ages are more likely to indicate state-related changes in depressive symptoms. This is also consistent with Hill et al. (2020) who found that the covariation between SMPs and depressive symptoms was strongest at later ages.

Greater SMPs at baseline predicted lower social function both initially and over time, consistent with previous cross-sectional findings (Kuiper et al., 2017). Surprisingly, chronic pain did not exacerbate this relationship, despite evidence that individuals with chronic pain tend to be more socially isolated (Cohen et al., 2010; Hirase et al., 2019; Penny et al., 1999). Although pain is known to induce avoidance behaviors that can extend to non-painful activities - including social engagement (Glogan et al., 2023; Glogan et al., 2022; Sofaer-Bennett et al., 2007; Valentinova et al., 2023; Weng et al., 2021) - its impact on social function appears to be largely established by baseline. Notably, increases in SMPs still correspond to further declines in social functioning in either group. This suggests that SMPs can still harm social functioning among people with chronic pain long-term, underscoring the need for targeted interventions to enhance social health in older adults with chronic pain. In particular, social engagement interventions (e.g., group activities, arts-based tasks, guided reminiscence) have shown preliminary promise to improve social health in older adults and mitigate other negative outcomes such as cognitive decline (Siette et al., 2025). Interventions focused on reframing negative memory beliefs have also shown to improve social activity participation in older adults (Farina et al., 2023).

Limitations must be acknowledged in interpreting these findings. Our definition of chronic pain, while widely used in epidemiological studies, was not based on clinical assessment, which may limit the generalizability of our results. However, previous research has shown that similar definitions yield comparable findings regarding cognitive decline and dementia risk (Bell et al., 2025; Bell et al., 2022a, 2022b; Khalid et al., 2020; Whitlock et al., 2017). Furthermore, we did not assess specific chronic pain subtypes, which could have provided more nuanced insights. Chronic pain phenotypes such as multisite chronic pain has been shown to have unique genetic and non-genetic influences (Gasperi et al., 2021). Future research should explore the differential impacts of pain subtypes on SMPs. Furthermore, our measures of functioning are extensive but have limitations. Some measures, such as the SF36 may lack score variability to capture subtle changes in function over time.

Overall, our study highlights the need for clinicians to consider SMPs and chronic pain act as additive and compounding risk factors for widespread functional decline in older adults. At their initial screening, SMPs cross-sectionally related to physical and psychosocial limitations, but chronic pain also independently contributed to these limitations. Over time, a higher level of SMPs, especially among people with chronic pain, related to decline in reasoning and self-rated physical function. Regardless of chronic pain status, changes in SMPs track with changes in functioning broadly. These findings suggest that clinicians and researchers should prioritize older adults with elevated SMPs, especially with chronic pain, for early, targeted interventions. Tailored approaches - such as cognitive training, physical activity programs, and social engagement strategies - may help mitigate the adverse effects on both mental and physical functioning, ultimately enhancing quality of life and delaying the onset of disability and cognitive impairment in this vulnerable population.

## Figures and Tables

**Table 1:** Descriptives of analytical sample by chronic pain status.

		Overall		No chronic pain		Chronic pain		Test statistic	*p*-value
						
		M (SD) / n(%)	n	M (SD) / n(%)	n	M (SD) / n(%)	n		
Age (years)	M (SD)	73.2 (5.66)	2365		1983		382	0.75	0.455
Education (years)	M (SD)	13.7 (2.68)	2365		1983		382	−3.83	<0.001
Marital status			2365		1983		382	−11	<0.001
	*n*(%)	1427 (60.3 %)		1167 (58.8 %)		260 (68.1 %)			
	*n*(%)	938 (39.6 %)		816 (41.1 %)		122 (31.9 %)			
Gender			2365		1983		382	−20.1	<0.001
Male	*n*(%)	632 (26.7 %)		566 (28.5 %)		66 (17.3 %)			
Female	*n*(%)	1735 (73.3 %)		1419 (71.5 %)		316 (82.7 %)			
Intervention group			2365		1983		382	−4.04	0.258
Control group	*n*(%)	592 (25.0 %)		503 (25.3 %)		89 (23.3 %)			
Memory training	*n*(%)	601 (25.4 %)		504 (25.4 %)		97 (25.4 %)			
Reasoning training	*n*(%)	585 (24.7 %)		476 (24.0 %)		109 (28.5 %)			
Speed of processing training	*n*(%)	589 (24.9 %)		502 (25.3 %)		87 (22.8 %)			
Race			2365		1983		382		
Other	*n*(%)	33 (1.4 %)		26 (1.3 %)		7 (1.8 %)		−8.06	0.018
Black	*n*(%)	558 (23.6 %)		489 (24.6 %)		69 (18.1 %)			
White	*n*(%)	1776 (75.0 %)		1470 (74.1 %)		306 (80.1 %)			
MFQ-GFF	M (SD)	25.6 (13.9)	2365	24.2 (13.9)	2365	29.6 (14.0)	2365	−4.36	<0.001
Memory	M (SD)	0.0618 (0.894)	2352	0.058 (0.899)	1971	0.083 (0.869)	381	0.521	0.603
Speed of processing	M (SD)	−0.081 (0.850)	2360	−0.088 (0.857)	1967	−0.044 (0.818)	381	0.941	0.347
Reasoning	M (SD)	0.080 (0.895)	2358	0.085 (0.902)	1978	0.053 (0.860)	380	−0.67	0.505
SF-36 physical function	M (SD)	70.9 (23.1)	2362	74.4 (21.3)	1980	52.9 (23.6)	382	−16.5	<0.001
Turn360 (# of steps)	M (SD)	6.62 (1.80)	2060	6.53 (1.76)	1735	7.07 (1.97)	325	4.54	<0.001
Grip strength	M (SD)	24.5 (8.49)	2068	24.9 (8.52)	1767	22.6 (8.04)	301	−4.38	<0.001
CESD	M (SD)	4.97 (5.04)	2266	4.56 (4.82)	1906	7.13 (5.59)	360	8.14	<0.001
SF-36 social function	M (SD)	87.3 (19.4)	2365	89.4 (17.8)	1983	76.5 (23.4)	382	−10.2	<0.001

Note. CESD = Center for Epidemiological Studies Depression Scale; MFQ = Memory Function Questionnaire – Frequency of Forgetting [reversed scored so higher is more subjective memory problems]; SF-36 = Short-Form Quality of Life Survey; SMPs = subjective memory problems.

**Table 2:** Generalized linear model assessing the association of subjective memory problems, chronic pain, and covariates with cognitive function composite outcomes at baseline and over time.

	Memory			Reasoning			Speed		
			
Predictors	β	*CI*	*p*	β	*CI*	*p*	β	*CI*	*p*
(Intercept)	0.05	−0.04 to 0.13	0.296	0.05	−0.03 to 0.12	0.209	−0.04	−0.12 to 0.04	0.343
Baseline associations									
Chronic pain	0.01	−0.08 to 0.10	0.818	−0.02	−0.10 to 0.05	0.569	−0.03	−0.12 to 0.05	0.479
Baseline SMP	−0.47	−0.64 to −0.40	**<0.001**	−0.52	−0.75 to −0.29	**<0.001**	−0.23	−0.44 to −0.01	**0.037**
Chronic pain*baseline SMP	−0.11	−0.45 to 0.22	0.511	−0.23	−0.68 to 0.22	0.314	−0.09	−0.51 to 0.33	0.678
Prospective associations									
Chronic pain*time	−0.01	−0.04 to 0.02	0.41	−0.06	−0.10 to −0.01	**0.008**	0.001	−0.03 to 0.04	0.823
Baseline SMP*time	−0.01	−0.02 to 0.01	0.223	−0.10	−0.12 to −0.08	**0**	0.001	−0.02 to 0.02	0.863
Chronic pain*baseline SMP*time	−0.01	−0.04 to 0.02	0.514	−0.05	−0.09 to −0.01	**0.039**	−0.01	−0.04 to 0.03	0.769
Concurrent associations									
Time-varying SMP	−0.28	−0.44 to −0.12	**0.001**	−0.38	−0.60 to −0.15	**0.001**	−0.13	−0.34 to 0.07	0.208
Time-varying SMP*CP	−0.05	−0.38 to 0.27	0.738	−0.23	−0.66 to 0.21	0.31	−0.03	−0.43 to 0.38	0.902
Covariates									
Time	−0.11	−0.14 to −0.09	**<0.001**	0.11	0.08 to 0.15	**<0.001**	−0.04	−0.07 to −0.01	**0.004**
Baseline age	−0.38	−0.42 to −0.34	**<0.001**	−0.27	−0.30 to −0.24	**<0.001**	−0.41	−0.45 to −0.38	**<0.001**
Marital status (yes)	−0.19	−0.27 to −0.11	**<0.001**	−0.04	−0.11 to 0.03	0.224	0.04	−0.03 to 0.12	0.268
Race (Black)	−0.43	−0.52 to −0.34	**<0.001**	−0.56	−0.63 to −0.48	**<0.001**	−0.37	−0.45 to −0.28	**<0.001**
Race (other)	0.02	−0.28 to 0.32	0.901	0.01	−0.25 to 0.27	0.957	0.09	−0.20 to 0.39	0.535
Education	0.16	0.12 to 0.19	**<0.001**	0.25	0.21 to 0.28	**<0.001**	0.08	0.05 to 0.12	**<0.001**
CESD total	−0.03	−0.05 to −0.01	**0.004**	−0.06	−0.08 to −0.04	**<0.001**	−0.01	−0.03 to 0.01	0.384
Memory training	0.17	0.06 to 0.27	**0.002**	0.01	−0.08 to 0.10	0.843	0.01	−0.09 to 0.11	0.895
Reasoning training	0.01	−0.10 to 0.11	0.861	0.20	0.12 to 0.29	**<0.001**	0.06	−0.04 to 0.16	0.253
Speed training	−0.02	−0.12 to 0.09	0.745	−0.05	−0.13 to 0.04	0.294	0.34	0.24 to 0.44	**<0.001**
Memory training*time	0.02	−0.02 to 0.05	0.278	0.02	−0.03 to 0.06	0.494	−0.03	−0.07 to 0.01	0.138
Reasoning training*time	−0.03	−0.06 to 0.01	0.112	0.12	0.07 to 0.16	**<0.001**	0.01	−0.03 to 0.05	0.604
Speed training*time	−0.01	−0.04 to 0.02	0.532	−0.05	−0.10 to −0.01	**0.024**	0.10	0.06 to 0.14	**<0.001**

Note. CESD = Center for Epidemiological Studies Depression Scale; CP = chronic pain; SMPs = subjective memory problems. Bolded values indicate significant *p*-values at less than 0.05.

**Table 3:** Generalized linear model assessing the association of subjective memory problems, chronic pain, and covariates with physical function outcomes at baseline and over time.

	Self-rated physical function			Turn360			Grip Strength		
			
Predictors	β	*CI*	*p*	β	*CI*	*p*	β	*CI*	*p*
(Intercept)	0.21	0.13 to 0.30	**<0.001**	0.05	−0.04 to 0.14	0.263	−0.44	−0.56 to −0.31	**<0.001**
Baseline associations									
Chronic pain	−0.94	−1.03 to −0.86	**<0.001**	0.24	0.15 to 0.34	**<0.001**	−0.15	−0.30 to −0.01	**0.032**
Baseline SMP	−0.49	−0.67 to −0.31	**<0.001**	0.03	−0.24 to 0.29	0.856	−0.46	−0.82 to −0.10	**0.012**
Chronic pain*baseline SMP	−0.13	−0.49 to 0.24	0.498	0.02	−0.53 to 0.56	0.947	−0.19	−0.90 to 0.52	0.603
Prospective associations									
Chronic pain*time	0.02	−0.01 to 0.05	0.212	−0.06	−0.11 to −0.02	**0.010**	0.09	−0.03 to 0.21	0.13
Baseline SMP*time	−0.02	−0.03 to −0.01	**0.008**	0.03	0.01 to 0.05	**0.023**	−0.10	−0.15 to −0.04	**<0.001**
Chronic pain*baseline SMP*time	−0.05	−0.09 to −0.02	**0.001**	−0.01	−0.06 to 0.04	0.748	−0.01	−0.14 to 0.12	0.901
Concurrent associations									
Time-varying SMP	−0.35	−0.52 to −0.18	**<0.001**	−0.001	−0.26 to 0.26	0.982	−0.37	−0.72 to −0.03	**0.033**
Time-varying SMP*CP	−0.18	−0.53 to 0.16	0.303	−0.03	−0.56 to 0.49	0.897	−0.24	−0.91 to 0.44	0.494
Covariates									
Time	−0.19	−0.22 to −0.16	**<0.001**	0.25	0.21 to 0.29	**<0.001**	−0.27	−0.36 to −0.17	**<0.001**
Baseline age	−0.18	−0.22 to −0.14	**<0.001**	0.28	0.24 to 0.33	**<0.001**	−0.13	−0.18 to −0.08	**<0.001**
Marital status (yes)	0.14	0.07 to 0.22	**<0.001**	−0.03	−0.12 to 0.05	0.403	0.58	0.48 to 0.68	**<0.001**
Race (Black)	−0.19	−0.28 to −0.09	**<0.001**	0.03	−0.05 to 0.12	0.443	0.25	0.14 to 0.37	**<0.001**
Race (other)	0.001	−0.32 to 0.31	0.986	−0.26	−0.62 to 0.09	0.149	0.06	−0.33 to 0.44	0.780
Education	0.05	0.02 to 0.09	**0.005**	−0.04	−0.08 to 0.00	0.08	0.08	0.03 to 0.13	**0.001**
CESD total	−0.11	−0.13 to −0.09	**<0.001**	0.04	0.01 to 0.07	**0.004**	−0.04	−0.08 to −0.01	**0.026**
Memory training	−0.03	−0.13 to 0.07	0.601	−0.05	−0.15 to 0.06	0.402	0.11	−0.04 to 0.26	0.159
Reasoning training	−0.04	−0.14 to 0.06	0.411	−0.06	−0.17 to 0.05	0.258	0.20	0.05 to 0.36	**0.011**
Speed training	0.03	−0.07 to 0.13	0.576	−0.01	−0.12 to 0.09	0.815	0.20	0.04 to 0.35	**0.013**
Memory training*time	−0.02	−0.06 to 0.01	0.197	−0.02	−0.08 to 0.03	0.444	0.16	0.03 to 0.29	**0.016**
Reasoning training*time	0.01	−0.03 to 0.04	0.777	−0.05	−0.11 to 0.00	0.064	0.20	0.07 to 0.34	**0.004**
Speed training*time	0.03	−0.00 to 0.07	0.078	−0.06	−0.11 to 0.00	0.051	0.22	0.09 to 0.35	**0.001**

Note. CESD = Center for Epidemiological Studies Depression Scale; CP = chronic pain; SMPs = subjective memory problems. Bolded values indicate significant p-values at less than 0.05.

**Table 4:** Generalized linear model assessing the association of subjective memory problems, chronic pain, and covariates with psychosocial outcomes at baseline and over time.

	Social function			CESD		
		
Predictors	β	*CI*	*p*	β	*CI*	*p*
(Intercept)	0.15	0.08 to 0.21	**<0.001**	−0.08	−0.16 to 0.00	0.055
Baseline associations						
Chronic pain	−0.55	−0.62 to −0.48	**<0.001**	0.51	0.43 to 0.59	**<0.001**
Baseline SMP	−0.34	−0.58 to −0.11	**0.004**	1.01	0.79 to 1.24	**<0.001**
Chronic pain*baseline SMP	−0.41	−0.88 to 0.06	0.087	0.29	−0.16 to 0.73	0.202
Prospective associations						
Chronic pain*time	0.01	−0.03 to 0.05	0.625	0.001	−0.04 to 0.03	0.818
Baseline SMP*time	−0.02	−0.04 to −0.001	**0.027**	−0.001	−0.02 to 0.02	0.693
Chronic pain*baseline SMP*time	−0.01	−0.04 to 0.05	0.861	−0.04	−0.08 to 0.01	0.107
Concurrent longitudinal associations						
Time-varying SMP	−0.26	−0.49 to −0.03	**0.026**	0.8	0.58 to 1.02	**<0.001**
Time-varying SMP*CP	−0.28	−0.73 to 0.18	0.233	0.21	−0.22 to 0.64	0.331
Covariates						
Time	−0.09	−0.12 to −0.05	**<0.001**	0.08	0.05 to 0.12	**<0.001**
Baseline age	−0.06	−0.09 to −0.03	**<0.001**	0.06	0.02 to 0.10	**0.001**
Marital status (yes)	−0.01	−0.07 to 0.05	0.680	−0.04	−0.11 to 0.03	0.279
Race (Black)	−0.06	−0.13 to 0.01	0.076	−0.07	−0.16 to 0.01	0.089
Race (other)	0.05	−0.20 to 0.29	0.707	−0.12	−0.40 to 0.17	0.422
Education	−0.01	−0.03 to 0.02	0.691	−0.10	−0.13 to −0.06	**<0.001**
CESD total	−0.35	−0.38 to −0.33	**<0.001**			
Memory training	0.03	−0.04 to 0.11	0.389	0.01	−0.08 to 0.11	0.811
Reasoning training	0.02	−0.05 to 0.10	0.529	0.06	−0.03 to 0.16	0.206
Speed training	0.04	−0.04 to 0.11	0.370	0.01	−0.09 to 0.10	0.891
Memory training*time	−0.03	−0.08 to 0.02	0.223	−0.03	−0.07 to 0.02	0.223
Reasoning training*time	−0.01	−0.06 to 0.04	0.712	−0.01	−0.06 to 0.03	0.540
Speed training*time	0.001	−0.05 to 0.05	0.989	−0.05	−0.09 to −0.00	**0.038**

Note. CESD = Center for Epidemiological Studies Depression Scale; CP = chronic pain; SMPs = subjective memory problems. Bolded values indicate significant p-values at less than 0.05.

## Data Availability

Data relevant to the publication are publicly accessible (https://www.icpsr.umich.edu/web/NACDA/studies/36036) as are analytical code (https://github.com/trbellucsd/ACTIVE_Pain_Subjective_Memory).
